# Development, Characterization, and Application of Two Reporter-Expressing Recombinant Zika Viruses

**DOI:** 10.3390/v12050572

**Published:** 2020-05-22

**Authors:** Sang-Im Yun, Byung-Hak Song, Michael E. Woolley, Jordan C. Frank, Justin G. Julander, Young-Min Lee

**Affiliations:** 1 Department of Animal, Dairy, and Veterinary Sciences, College of Agriculture and Applied Sciences, Utah State University, Logan, UT 84322, USA; sangim.yun@usu.edu (S.-I.Y.); byunghak.song@aggiemail.usu.edu (B.-H.S.); michaeleverettwoolley@gmail.com (M.E.W.); jc.frank@aggiemail.usu.edu (J.C.F.); justin.julander@usu.edu (J.G.J.); 2 Institute for Antiviral Research, Utah State University, Logan, UT 84322, USA; 3 Veterinary Diagnostics and Infectious Diseases, Utah Science Technology and Research, Utah State University, Logan, UT 84341, USA

**Keywords:** Zika virus, infectious cDNA, reporter-expressing virus, green fluorescent protein, luciferase, viral protein expression profile, antiviral

## Abstract

Zika virus (ZIKV), a mosquito-borne transplacentally transmissible flavivirus, is an enveloped virus with an ~10.8 kb plus-strand RNA genome that can cause neurological disease. To facilitate the identification of potential antivirals, we developed two reporter-expressing ZIKVs, each capable of expressing an enhanced green fluorescent protein or an improved luminescent NanoLuc luciferase. First, a full-length functional ZIKV cDNA clone was engineered as a bacterial artificial chromosome, with each reporter gene under the cap-independent translational control of a cardiovirus-derived internal ribosome entry site inserted downstream of the single open reading frame of the viral genome. Two reporter-expressing ZIKVs were then generated by transfection of ZIKV-susceptible BHK-21 cells with infectious RNAs derived by in vitro run-off transcription from the respective cDNAs. As compared to the parental virus, the two reporter-expressing ZIKVs grew to lower titers with slower growth kinetics and formed smaller foci; however, they displayed a genome-wide viral protein expression profile identical to that of the parental virus, except for two previously unrecognized larger forms of the C and NS1 proteins. We then used the NanoLuc-expressing ZIKV to assess the in vitro antiviral activity of three inhibitors (T-705, NITD-008, and ribavirin). Altogether, our reporter-expressing ZIKVs represent an excellent molecular tool for the discovery of novel antivirals.

## 1. Introduction

Zika virus (ZIKV) is a mosquito-borne arbovirus belonging to the genus *Flavivirus*, family *Flaviviridae* [1]. Within the genus, ZIKV is closely related to other clinically important mosquito-borne flaviviruses, such as dengue (DENV), Japanese encephalitis (JEV), West Nile (WNV), and yellow fever (YFV) viruses, as well as several medically significant tick-borne flaviviruses, including tick-borne encephalitis and Powassan viruses [2]. In humans, ZIKV is spread horizontally by blood-sucking mosquitoes of the genus *Aedes* (e.g., *A. aegypti* and *A. albopictus*) and through sexual contact, but it can also be passed vertically from a pregnant woman to her fetus in utero [3,4,5,6,7,8]. While uncommon, ZIKV transmission can also occur via blood transfusion and breastfeeding [9,10]. The clinical outcome of ZIKV infection varies significantly, with the estimated prevalence of asymptomatic cases ranging from ~30% to >85% [11], depending on a combination of viral strain, study population, and environmental conditions at a given time [12,13,14]. Symptomatic ZIKV infection typically presents as a mild self-limiting illness with nonspecific flu-like symptoms [7,15], but it can also lead to a range of severe neurological disorders, such as Guillain-Barré syndrome (GBS) in adults and congenital Zika syndrome (CZS) in neonates [16,17,18,19,20]. However, neither effective drugs nor vaccines are available for the control of ZIKV infection.

ZIKV is an ~50 nm diameter particle that has an outer lipid bilayer embedded with 180 copies of each of the two well-defined surface proteins, membrane (M) and envelope (E) [21,22]. Inside the particle is an ill-defined nucleocapsid formed by the association of multiple copies of the capsid (C) protein with one copy of the viral genome [23]. The ZIKV genome is a linear, single-stranded, positive-sense RNA of approximately 10,800 nucleotides (nt) [24] and contains a 7-methylguanosine (m^7^G) cap at the 5′ end, but no poly(A) tail at the 3′ end [25]. It has a single long open reading frame (ORF) flanked by the two short highly-structured untranslated regions (5′ and 3′ UTRs) that play key regulatory roles in viral translation and RNA replication through 5′–3′ long-distance RNA-RNA interactions [1,26]. The ORF encodes a large polyprotein that is processed by both host cell- and virus-encoded proteases to produce at least 10 major proteins: three structural (C, prM [precursor of M], and E) and seven nonstructural (NS1, NS2A, NS2B, NS3, NS4A, NS4B, and NS5) [12]. The viral genomic RNA serves not only as a template for both translation and RNA replication, but also as a substrate for degradation by cellular 5′ to 3′ exoribonucleases that stops at a higher-order RNA structure within the 3′UTR, thereby producing small non-coding RNAs [27,28]. These non-coding RNAs counteract host cell antiviral responses [29].

ZIKV and other flaviviruses share a similar replication strategy [1]. Once bound to the cell surface, the virion is taken up by viral protein E-mediated endocytosis [30]. The internalized virion travels to an endosomal compartment where the acidic environment induces large conformational changes in the E proteins, leading to fusion of the viral membrane with the endosomal membrane and release of the viral genomic RNA into the cytoplasm [31,32]. After initial translation of the genomic RNA, viral RNA replication takes place inside virus-induced, specialized membranous structures derived from the endoplasmic reticulum (ER) [33] and is catalyzed by two bidomain multifunctional proteins: (i) NS3, comprising a serine protease domain (requiring NS2B as a cofactor) and an RNA helicase/NTPase/RTPase domain [34,35,36,37,38] and (ii) NS5, comprising a methyltransferase/guanylyltransferase domain and an RNA-dependent RNA polymerase domain [39,40,41,42,43,44]. Viral assembly is initiated by budding of the viral genomic RNA and C proteins into the ER lumen to incorporate the prM and E proteins into a budding immature particle [45]. The immature virion then usurps the cellular secretory pathway to exit the cell [46]; during this process, the prM proteins are processed into the mature M proteins, followed by major structural rearrangements in the M and E proteins prior to viral release from the cell [47].

The ancestral prototype strain of ZIKV designated MR-766 was first isolated from a naturally infected symptomatic rhesus macaque in the Zika forest of Uganda in 1947 [48]. Over the half-century following its discovery, ZIKV circulated only in the tropical and subtropical regions of two connected continents, Africa and Asia, without posing a significant threat to humans [7,49]. In 2007, however, it first emerged outside Africa and Asia, causing a relatively small but historically important outbreak in Yap Island in the western North Pacific, with ~5000 people estimated to be infected [15,50,51]. From there, the virus moved eastward across the Pacific Ocean, causing a large outbreak of an estimated ~30,000 infections in French Polynesia and other nearby islands in the central South Pacific in 2013–2014 [52,53,54], and then a massive outbreak of an estimated >200,000 infections in the Americas and their adjacent islands in 2015–2016 [55,56,57]. While the Yap Island outbreak was characterized by a mild illness with fever, rash, arthralgia, and/or conjunctivitis, the two French Polynesian and American outbreaks were the first to reveal an association between ZIKV infection and severe neurological complications, such as GBS and CZS [7,58,59]. Because of its impact on global public health, ZIKV is now a priority pathogen for the development of therapeutics and vaccines.

In the present study, we created two reporter-expressing ZIKVs to provide an ideal toolkit for monitoring viral replication with no need for secondary methodologies in order to facilitate the screening and testing of antiviral drug candidates. In each case, we engineered a full-length infectious cDNA clone of ZIKV MR-766 to insert a reporter expression cassette composed of an enhanced green fluorescent protein (eGFP) or improved luminescent NanoLuc luciferase (nLUC) gene and a cardiovirus-derived internal ribosome entry site (IRES) controlling its expression. We recovered two reporter-expressing ZIKVs from the respective cDNAs, examined their biological properties, and used them to evaluate the in vitro antiviral activity of three inhibitors. Our reporter-expressing ZIKVs provide a new means not only for the discovery of therapeutics, but also for the study of viral replication.

## 2. Materials and Methods

### 2.1. Cell Culture

BHK-21 cells were cultured as monolayers in α-modified minimal essential medium (MEM) containing 10% fetal bovine serum (FBS), 2 mM L-glutamine, 1 × MEM vitamin solution, and 10 U/mL penicillin-streptomycin at 37 °C with 5% CO_2_ in a humidified incubator [60]. All cell culture reagents were purchased from Gibco-Life Technologies, Carlsbad, CA, USA.

### 2.2. DNA Cloning

We constructed two reporter-encoding full-length cDNA clones (designated pZIKV-eGFP and pZIKV-nLUC) of ZIKV MR-766, using our previously generated infectious cDNA clone pBac/MR-766 (here called pZIKV) as the parental construct [12]. The two cDNA clones were engineered by standard recombinant DNA techniques and were finally purified by CsCl banding. The nucleotide sequence of the PCR-amplified regions was verified by sequencing and the overall integrity of the full-length cDNA clones was confirmed by extensive restriction mapping. The oligonucleotide primers used in this study are summarized in Table 1.

First, pZIKV-nLUC was constructed initially by amplifying two cDNA fragments of pZIKV by PCR. The first amplicon (PCR I), produced using primers ORF3f and ORF3r, was the 200-bp fragment comprising the 3′-terminal region of the viral ORF, followed by the 5′ end portion of the viral 3′UTR. The second amplicon (PCR II), produced using primers 3UTRf and 3UTRr, was the 483-bp fragment spanning the entire viral 3′UTR. In addition, the 1113-bp cDNA fragment containing the encephalomyocarditis virus (EMCV) IRES-driven nLUC expression cassette was obtained from pJEV-nLUC (Yun, SI and Lee, YM; unpublished) by digestion with *Eco*RI and *Nsi*I. The 188-bp *Rsr*II-*Eco*RI fragment of PCR I was then subcloned into pRS2, along with the 1113-bp *Eco*RI-*Nsi*I fragment of the nLUC expression cassette by using *Rsr*II and *Nsi*I, thereby producing pRS/Luc-1. Next, the 471-bp *Nsi*I-*Sac*II fragment of PCR II was inserted downstream of the EMCV IRES-driven nLUC expression cassette in pRS/Luc-1 using the same two restriction enzymes, thereby producing pRS/Luc-2. Finally, the 1735-bp *Bam*HI-*Bsi*WI fragment of pRS/Luc-2 was ligated with the 17971-bp *Bsi*WI-*Bam*HI fragment of pZIKV, creating pZIKV-nLUC. For the replication-incompetent pZIKV-nLUC^POL−^ mutant, in which the catalytic site of the NS5 polymerase domain was inactivated by site-directed mutagenesis (changing GDD to GAA), two overlapping cDNA fragments were first amplified by PCR with two primer pairs, FORf + POLr and POLf + REVr. The resulting two fragments were then fused by a second round of PCR with the outer primers FORf and REVr. Finally, the 1372-bp *Mlu*I-*Bam*HI fragment of the resulting amplicons was ligated with the 18334-bp *Bam*HI-*Mlu*I fragment of pZIKV-nLUC, creating pZIKV-nLUC^POL−^.

For the construction of pZIKV-eGFP, we first amplified a 786-bp cDNA fragment corresponding to the complete coding sequence of eGFP from pJEV-eGFP [60] by PCR, using primers EGFPf and EGFPr. The 521-bp nLUC ORF was cut out from pRS/Luc-2 by digestion with *Nco*I and *Nsi*I and replaced by ligating the 773-bp *Nco*I-*Nsi*I fragment of the eGFP amplicon in its place, thereby producing pRS/eGFP. As was done for the construction of pZIKV-nLUC, the 1987-bp *Bam*HI-*Bsi*WI fragment of pRS/eGFP was finally ligated with the 17971-bp *Bsi*WI-*Bam*HI fragment of pZIKV, creating pZIKV-eGFP.

### 2.3. In Vitro Transcription

Run-off transcription was carried out to synthesize full-length ZIKV RNAs in vitro from a cloned cDNA of pZIKV or its derivatives [12,61]. In short, each full-length ZIKV cDNA clone was first linearized by digestion with *Psr*I (SibEnzyme, West Roxbury, MA, USA). In all cases, ~200 ng of the linearized cDNA template was mixed in a 25-μL transcription reaction with 20 U SP6 RNA polymerase, 0.6 mM cap analog m^7^GpppA, 1 mM each NTP, 10 mM dithiothreitol, 40 U RNaseOUT, and the buffer supplied by the manufacturer (New England Biolabs, Ipswich, MA, USA). The reactions were first allowed to proceed at 37 °C for 1 h and further incubated for an additional 30 min with 10 U DNase I to degrade the template cDNA. RNA transcripts were quantified by adding 0.5 μM [^3^H]UTP (PerkinElmer, Boston, MA, USA) per reaction and calculating the amount of its incorporation into RNA based on the binding of RNA to DE81 paper (Whatman, Maidstone, United Kingdom). The integrity of the RNA transcripts was examined by running an aliquot of each reaction on a 0.6% agarose gel and visualizing the bands by ethidium bromide staining.

### 2.4. RNA Transfection

BHK-21 cells were transfected with in vitro-transcribed RNAs using the ECM 830 electroporation system (BTX, San Diego, CA, USA) as reported previously [61,62]. In brief, subconfluent cell monolayers were trypsinized to yield a single-cell suspension, washed three times with cold phosphate-buffered saline (PBS), and then resuspended in cold PBS at 2 × 10^7^ cells/mL. A 400-μL aliquot of the cell suspension was mixed with 2 μg of RNA on ice. The RNA-cell mixture was then transferred to a 2-mm-gap cuvette (BTX) and immediately pulsed five times at 980 V with a 99-μs pulse length. The electroporated cells were diluted to 1 mL with complete cell culture medium and kept at room temperature for 10 min to recover from the electric pulses.

### 2.5. Infectious Center Assay

An infectious center assay was used to determine the specific infectivity of run-off RNA transcripts derived from pZIKV, pZIKV-eGFP, or pZIKV-nLUC. First, BHK-21 cells were transfected with in vitro-transcribed RNAs as described above, serially diluted 1:10 in 1 mL of complete cell culture medium, and placed on naïve BHK-21 cells grown in a 6-well plate (5 × 10^5^ cells/well). Cells were allowed to attach to the plates for 6 h, after which the culture medium was replaced with an agar overlay composed of 0.5% SeaKem LE agarose and 10% FBS in MEM. Plates were then incubated at 37 °C with 5% CO_2_ for 4 days before the infectious foci were visualized by immunostaining [62]. Specifically, cells were first fixed with 7% formaldehyde in PBS overnight, followed by three washes (10 min each) with PBS. The fixed cells were then permeabilized with 0.25% Triton X-100 in PBS for 10 min and incubated for 2 h with a 1:1000 dilution of a polyclonal rabbit α-NS3 antiserum [12]. After three washes with PBS, the primary antibody-treated cells were again incubated for 2 h with a 1:1000 dilution of horseradish peroxidase-conjugated goat α-rabbit IgG (Jackson ImmunoResearch Laboratories, West Grove, PA, USA). The secondary antibody-labeled cells were then washed three times with PBS before being developed by incubation with 3,3′-diaminobenzidine (Vector Laboratories, Burlingame, CA, USA).

### 2.6. Immunoblot Analysis

A series of immunoblot analyses was employed to compare the overall viral protein expression profile generated from the infectious RNAs derived from pZIKV, pZIKV-eGFP, or pZIKV-nLUC: BHK-21 cells were transfected with in vitro-synthesized RNAs and seeded in a 6-well plate at a density of 3 × 10^5^ cells/well. Every day for 4 days, cells were harvested with 200 μL per well of cell lysis buffer (80 mM Tris-HCl [pH 6.8], 2% sodium dodecyl sulfate [SDS], 100 mM dithiothreitol, 10% glycerol, and 0.2% bromophenol blue). Equal volumes of whole cell lysates were boiled for 5 min and separated by SDS-polyacrylamide gel electrophoresis (PAGE). Resolved proteins on the gel were blotted electrophoretically onto a polyvinylidene difluoride membrane using a Trans-Blot SD Semi-Dry Transfer Cell (Bio-Rad Laboratories, Hercules, CA, USA). After blotting, the membrane was blocked for 1 h in wash buffer (0.2% Tween 20 in PBS) with 5% non-fat dry milk and washed three times (all washes 10 min each). The blocked membrane was then probed for a protein of interest for 2 h in staining buffer (0.5% non-fat dry milk in wash buffer), with one of the following polyclonal rabbit antisera at the indicated dilution [12,63]: α-C (1:100), α-M (1:1000), α-E (1:250), α-NS1 (1:1000), α-NS2B (1:100), α-NS3 (1:1000), α-NS4A (1:1000), α-NS4B (1:1000), α-NS5 (1:1000), and α-GAPDH (1:5000). The primary antibody-treated membrane was washed three times, followed by a 2-h incubation in staining buffer with a 1:1000 dilution of alkaline phosphatase-conjugated goat α-rabbit IgG (Jackson ImmunoResearch Laboratories). The secondary antibody-labeled membrane was washed three times with wash buffer and rinsed once with PBS. Finally, the membrane was incubated in a developing solution containing 5-bromo-4-chloro-3-indolyl-phosphate and nitroblue tetrazolium (MilliporeSigma, St. Louis, MO, USA).

### 2.7. Fluorescence Microscopy

The expression of eGFP was analyzed in BHK-21 cells both after the transfection of cDNA-derived viral RNAs and after the infection of cDNA-derived recombinant viruses. In the first case, cells were electroporated with 2 μg of viral RNAs, then plated in a 4-well chamber slide (1 × 10^5^ cells/well) and incubated for 40 h. In the second case, cells were first seeded in a 4-well chamber slide (1 × 10^5^ cells/well) for 12 h, then infected with each of recombinant viruses at a multiplicity of infection (MOI) of 1 and incubated for 40 h. In both cases, the cells were fixed for 10 min with 4% formaldehyde in PBS, washed twice with PBS, and mounted in an anti-fading solution (Dako, Glostrup, Denmark). Fluorescence images were recorded with an LSM-710 confocal microscope (Carl Zeiss, Jena, Germany).

### 2.8. Luciferase Assay

The activity of nLUC was examined in BHK-21 cells both after the transfection of cDNA-derived viral RNAs and after the infection of cDNA-derived recombinant viruses. For the transfection experiments, cells were electroporated with 2 μg of viral RNAs, then split into a 6-well plate (3 × 10^5^ cells/well) and incubated for 4 days. For the infection experiments, cells were first placed in a 6-well plate (3 × 10^5^ cells/well) for 12 h, then infected with each of recombinant viruses at a MOI of 1 and incubated for 4 days. In both types of experiment, the nLUC assay was performed according to the protocol from the manufacturer of the Nano-Glo Luciferase Assay (Promega, Madison, WI, USA). In brief, cells were harvested using 200 μL per well of Assay Buffer at various time points throughout incubation. The nLUC assay was performed by combining 10 μL of each cell lysate with 10 μL of Assay Buffer and 20 μL of Assay Substrate in a 96-well plate. Luminescence readings were obtained with a Synergy HT microplate reader (BioTek Instruments, Winooski, VT, USA).

### 2.9. Antiviral Testing

Three compounds used in this study were: (i) T-705, dissolved in dimethyl sulfoxide (DMSO) to make a stock solution of 20 mg/mL and tested at final concentrations of 0 (DMSO alone), 1, 4, 20, and 100 μg/mL; (ii) NITD-008, dissolved in DMSO to make a stock solution of 10 mM and tested at final concentrations of 0 (DMSO alone), 1, 4, 20, and 100 μM; and (iii) ribavirin, dissolved in cell culture medium to make a stock solution of 2 mg/mL and tested at final concentrations of 0, 8, 40, 200, and 1000 μg/mL. To examine the cell toxicity of the compounds, BHK-21 cells were placed in a 96-well plate (1 × 10^4^ cells/well) and cultivated for 48 h in 100 μL of complete cell culture medium without or with one of the compounds at the concentrations indicated. Cell toxicity was monitored using the CellTiter-Blue Cell Viability Assay (Promega) by adding 20 μL per well of CellTiter-Blue Reagent, incubating the plate at 37 °C with 5% CO_2_ for 30 min, and then recording fluorescence readings with a Synergy HT microplate reader (BioTek Instruments) equipped with a 560-nm excitation filter and a 590-nm emission filter. To analyze the antiviral activity of the compounds against ZIKV infection in vitro, BHK-21 cells were seeded into a 96-well plate (1 × 10^4^ cells/well) for 12 h, infected with rZIKV-nLUC at a MOI of 0.1 for 1 h, and then incubated for 48 h in 100 μL of complete cell culture medium in the absence or presence of a compound at the concentrations indicated. At the end of the incubation, antiviral activity was evaluated by measuring both the level of extracellular virus production in culture supernatants using the immunofocus assay reported previously [12] and the level of intracellular nLUC expression in cell lysates using the luciferase assay described above.

## 3. Results

### 3.1. Construction of Two Reporter-Encoding Full-Length ZIKV cDNA Clones

We selected two reporters for the development of reporter-expressing ZIKVs: an enhanced green fluorescent protein (eGFP) and an improved luminescent NanoLuc luciferase (nLUC). They each have particular attributes that make them uniquely valuable: (1) eGFP (~29 kDa) is a genetically engineered variant of the original jellyfish-derived GFP that is codon-optimized for higher protein expression in mammalian cells and contains chromophore mutations for brighter fluorescence [64,65]. The newly acquired properties of eGFP, together with its intrinsic fluorescence in the absence of any substrate or cofactor, make it a convenient marker for visual monitoring of viral replication in a variety of mammalian cells. (2) nLUC (~19 kDa) is a small, ATP-independent, and thermostable luciferase that is brighter than the traditional Photinus or Renilla luciferase, with reduced background luminescence [66]. These unique properties of nLUC make it an attractive reporter for precise quantification of viral replication.

**Figure 1:**
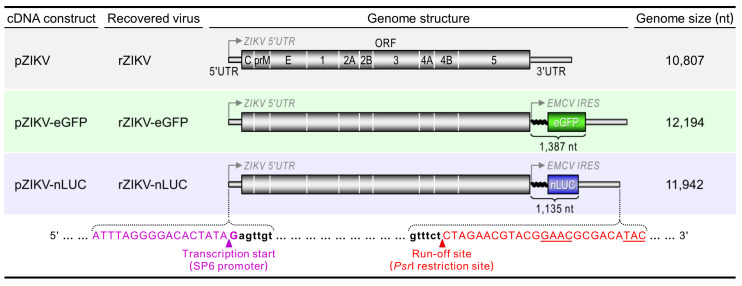
Schematic representation of two reporter-encoding full-length ZIKV cDNA clones. The top panel shows the genome structure of the parental ZIKV (rZIKV) recovered from its full-length cDNA clone (pZIKV). The two bottom panels depict the genome structures of two reporter-encoding ZIKVs (rZIKV-eGFP and rZIKV-nLUC) rescued from their respective full-length cDNA clones (pZIKV-eGFP and pZIKV-nLUC). In both constructs, each reporter gene driven by the encephalomyocarditis virus (EMCV) internal ribosome entry site (IRES) is inserted after the stop codon of the viral open reading frame (ORF) at the beginning of the 3′ untranslated region (UTR). The nucleotide sequences provided below the three full-length cDNA clones correspond to the 5′ SP6 promoter (magenta uppercase) and 3′ unique *Psr*I restriction site (red uppercase) engineered immediately upstream and downstream of the viral genome (black lowercase), respectively. Also, the arrowheads marked below the nucleotide sequences indicate the transcription start and run-off sites for in vitro transcription. The genome size of each recombinant virus is shown, as is the insert size of each reporter gene expression cassette.

As illustrated schematically in Figure 1, we constructed two new full-length ZIKV cDNA clones encoding eGFP (pZIKV-eGFP) or nLUC (pZIKV-nLUC), based on a single-copy bacterial artificial chromosome (BAC) vector, using our previously developed functional BAC clone of ZIKV MR-766 (pZIKV) [12]. The parent pZIKV contains a complete cDNA copy of the viral genomic RNA, flanked on each side by a 5′ SP6 promoter sequence and a 3′ *Psr*I restriction site for the production of run-off RNA transcripts with authentic 5′ and 3′ ends of the viral genome. We kept the single ZIKV ORF intact and under the cap-dependent translational control of its own 5′UTR as the first cistron and inserted a reporter expression cassette as the second cistron between the end of the viral ORF and the beginning of the viral 3′UTR. The reporter expression cassette consists of the coding sequence for eGFP (768 nt) or nLUC (516 nt) under the cap-independent translational control of a 619-nt IRES from EMCV. The insertion of such a reporter expression cassette therefore increases the genome size of recombinant ZIKVs (indicated by the prefix “r”) recoverable from their corresponding cDNAs: from 10,807 nt (rZIKV) to 12,194 nt (rZIKV-eGFP) or 11,942 nt (rZIKV-nLUC).

### 3.2. Production of Infectious RNA Transcripts from Two Reporter-Encoding Full-Length ZIKV cDNA Clones

**Figure 2:**
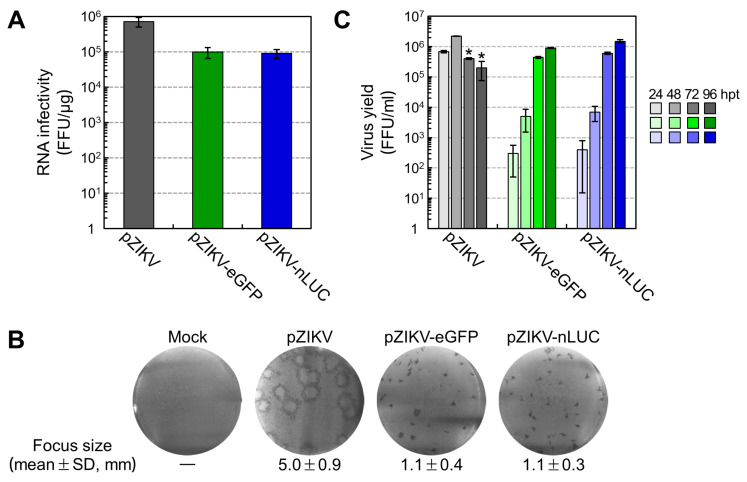
Recovery of two infectious reporter-encoding ZIKVs. BHK-21 cells were mock-transfected or transfected with 2 μg of run-off RNA transcripts synthesized in vitro from each of the three *Psr*I-linearized cDNA templates, namely pZIKV, pZIKV-eGFP, and pZIKV-nLUC. (**A**) RNA infectivity. The specific infectivity of run-off RNA transcripts was determined by infectious center assays involving immunostaining the infectious foci at 96 h post-transfection (hpt) with a rabbit α-NS3 antiserum. Data are presented as focus-forming units (FFU) per μg. (**B**) Focus morphology. Representative focus images are shown, with the average focus diameters (mean ± standard deviations [SD]) estimated by counting 20 randomly picked foci from two independent experiments. (**C**) Virus yield. The level of virus production was determined by immunofocus assays involving harvesting culture supernatants at the indicated time points after RNA transfection and titrating the amounts of infectious virions on naïve BHK-21 cells. Black single asterisks indicate the time points at which >90% of cells were detached from the culture plate as a result of virus-induced cell death. Error bars represent standard deviations of two independent experiments.

We assessed the specific infectivity of RNA transcripts derived from each of two reporter-encoding full-length ZIKV cDNA clones (pZIKV-eGFP and pZIKV-nLUC), as compared to those derived from the parent pZIKV. In the case of all three clones, RNAs were synthesized in vitro by run-off transcription of each *Psr*I-linearized cDNA template and equal amounts of these RNAs were transfected into ZIKV-susceptible BHK-21 cells for infectious center assays. At 96 h post-transfection (hpt), the infectious centers of foci were visualized by immunostaining with a rabbit α-NS3 antiserum. On average, the infectivity of pZIKV-derived RNAs was estimated to be 7.1 × 10^5^ focus-forming units (FFU) per μg, whereas the infectivities of the RNAs derived from both pZIKV-eGFP and pZIKV-nLUC were equally reduced by ~1 log to a range of 9.0–9.8 × 10^4^ FFU/μg (Figure 2A). With respect to focus morphology, there was an ~5-fold decrease in the size of the NS3-positive foci observed in cells transfected with RNAs derived from pZIKV-eGFP and pZIKV-nLUC, when compared to those in cells transfected with pZIKV-derived RNAs (Figure 2B).

We next determined the amount of infectious recombinant ZIKVs released into culture supernatants from RNA-transfected BHK-21 cells. Culture media were harvested daily for 4 days and virus yields were titrated by immunofocus assays on naïve BHK-21 cells using the rabbit α-NS3 antiserum. The parent rZIKV accumulated rapidly, achieving a peak titer of 2.2 × 10^6^ FFU/mL at 48 hpt, which then dropped over the next 48 h to 2.0 × 10^5^ FFU/mL at 96 hpt because of a lack of viable cells remaining (Figure 2C). On the other hand, both rZIKV-eGFP and rZIKV-nLUC accumulated at a nearly identically slow-but-steady rate for the entire 4 days, reaching their maximum titers of 0.9–1.5 × 10^6^ FFU/mL at 96 hpt (Figure 2C). This delay in virus production is in line with the observed decreases in RNA infectivity (Figure 2A) and focus size (Figure 2B). Altogether, these results indicate that RNA transcripts derived from each of our eGFP- and nLUC-encoding full-length ZIKV cDNA clones are equally functional, having a specific infectivity of ~10^5^ FFU/μg, about one log lower than those derived from the parental ZIKV cDNA clone (as a result of the increase in viral genome size).

### 3.3. Probing of Viral Protein Accumulation During ZIKV Genomic RNA Replication

**Figure 3:**
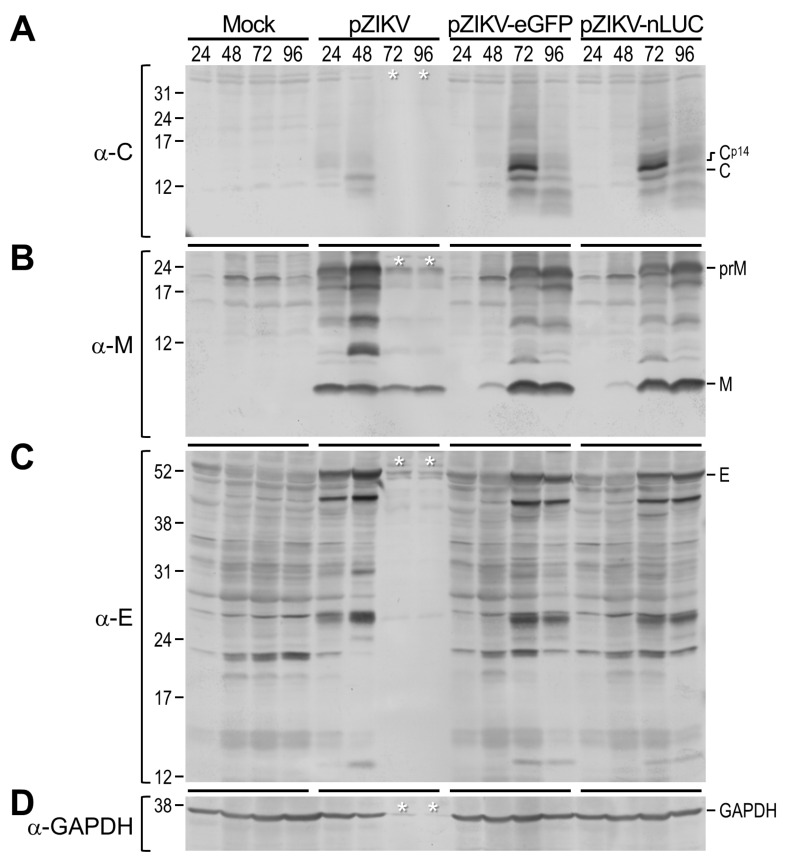
Profiling of viral structural protein expression. BHK-21 cells were mock-transfected or transfected with 2 μg of the infectious RNAs transcribed from pZIKV, pZIKV-eGFP, or pZIKV-nLUC. The cells were then lysed at 24, 48, 72, or 96 h after transfection. Equal amounts of total cell lysates were resolved by SDS-PAGE, followed by immunoblotting with one of the following three rabbit antisera specific to the viral structural proteins: α-C (**A**), α-M (**B**), or α-E (**C**). In parallel, a rabbit α-GAPDH antiserum was used as a cell lysate loading-control antibody (**D**). Protein molecular size markers in kDa are indicated on the left, and major viral structural proteins and GAPDH are marked on the right. White single asterisks indicate that >90% of cells were detached from the culture plate by virus-induced cell death.

**Figure 4:**
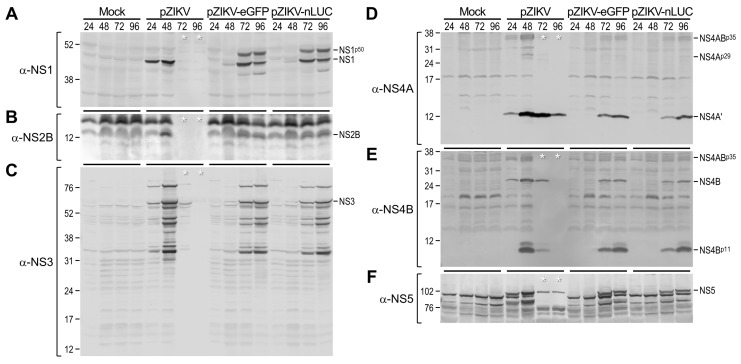
Profiling of viral nonstructural protein expression. BHK-21 cells were mock-transfected or transfected with 2 μg of the infectious RNAs synthesized from pZIKV, pZIKV-eGFP, or pZIKV-nLUC. The cells were then lysed at 24, 48, 72, or 96 h after transfection. Equal amounts of whole-cell lysates were separated by SDS-PAGE, followed by immunoblotting with one of the following six rabbit antisera specific to the viral nonstructural proteins: α-NS1 (**A**), α-NS2B (**B**), α-NS3 (**C**), α-NS4A (**D**), α-NS4B (**E**), or α-NS5 (**F**). Protein molecular size markers in kDa are indicated on the left and major viral nonstructural proteins are marked on the right. White single asterisks indicate that >90% of cells were detached from the culture plate by virus-induced cell death.

We compared the protein expression profile and accumulation kinetics of almost all the viral gene products made from the infectious RNA derived from each of our two reporter-encoding full-length ZIKV cDNA clones and from their parental cDNA clone for comparison. To this end, BHK-21 cells were mock-transfected or transfected with an equal amount of infectious RNA transcribed in vitro from pZIKV-eGFP, pZIKV-nLUC, or pZIKV. Following transfection, the cells were harvested daily for 4 days. Equal amounts of whole-cell lysates were then separated on polyacrylamide gels under denaturing and reducing conditions, followed by immunoblotting, with each of our nine previously well-characterized ZIKV-specific polyclonal rabbit antisera [12], i.e., a first set of three (α-C, α-M, and α-E) capable of detecting all of three viral structural proteins (Figure 3) and a second set of six (α-NS1, α-NS2B, α-NS3, α-NS4A, α-NS4B, and α-NS5) identifying six of the seven viral nonstructural proteins (all except NS2A) (Figure 4). Also, a rabbit α-GAPDH antiserum was used to detect the cellular housekeeping GAPDH protein as a sample loading control for our immunoblotting.

**Figure 5:**
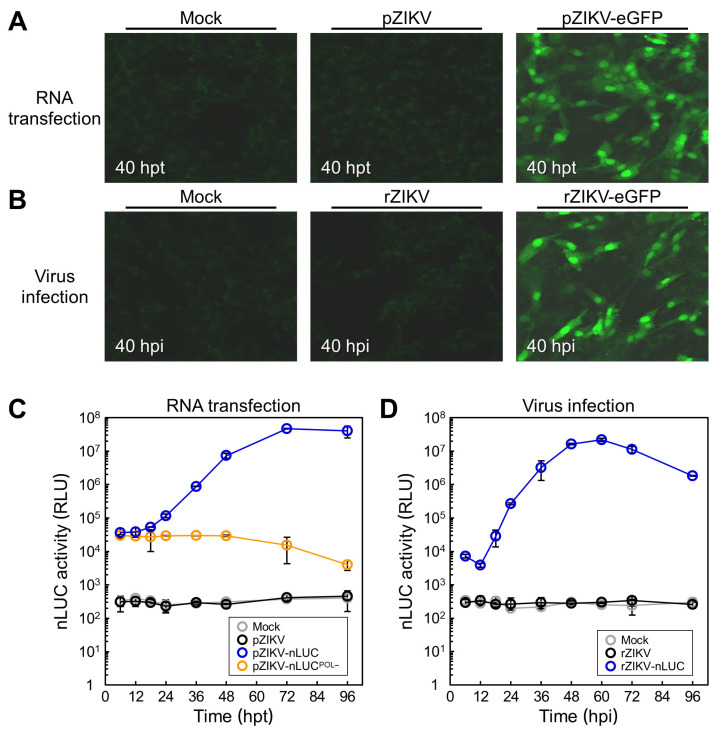
Monitoring of reporter gene expression. (**A**,**B**) eGFP expression. BHK-21 cells were mock-transfected or transfected with 2 μg of the in vitro-transcribed RNAs from pZIKV or pZIKV-eGFP (**A**). In parallel, BHK-21 cells were mock-infected or infected with rZIKV or rZIKV-eGFP at a multiplicity of infection (MOI) of 1 (**B**). At 40 h post-transfection (hpt) or post-infection (hpi), cells were fixed and examined by confocal microscopy. Images were acquired using a 20X dry lens (Plan-Apochromat 20X/0.8 M27). Representative fluorescent images from one of two experiments are presented. (**C**,**D**) nLUC activity. BHK-21 cells were mock-transfected or transfected with 2 μg of the in vitro-synthesized RNAs of pZIKV, pZIKV-nLUC, or pZIKV-nLUC^POL−^ (**C**). In addition, BHK-21 cells were mock-infected or infected with rZIKV or rZIKV-nLUC at a MOI of 1 (**D**). During the incubation period of 4 days, cells were lysed at the indicated time points after RNA transfection or virus infection for nLUC assays. The nLUC activity is expressed in relative light units (RLU). Error bars indicate standard deviations of two independent experiments.

**Figure 6:**
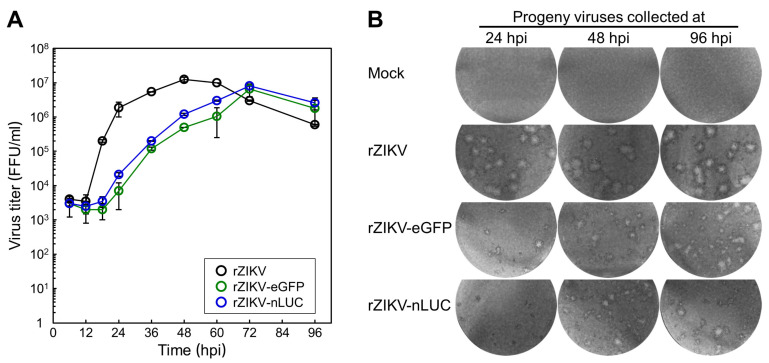
Characterization of viral growth and stability. BHK-21 cells were infected at a MOI of 1 with rZIKV, rZIKV-eGFP, or rZIKV-nLUC. (**A**) Viral growth. Culture supernatants were collected at the indicated time points after infection for 4 days to determine the accumulation level of infectious progeny virions by immunofocus assays on naïve BHK-21 cells. The data represent mean values, and the error bars show standard deviations. (**B**) Viral stability. Presented are representative images of the infectious foci formed by the infectious progeny virions that were harvested at 24, 48, or 96 h post-infection (hpi). Mock-infected cells were included as a negative control.

**Figure 7:**
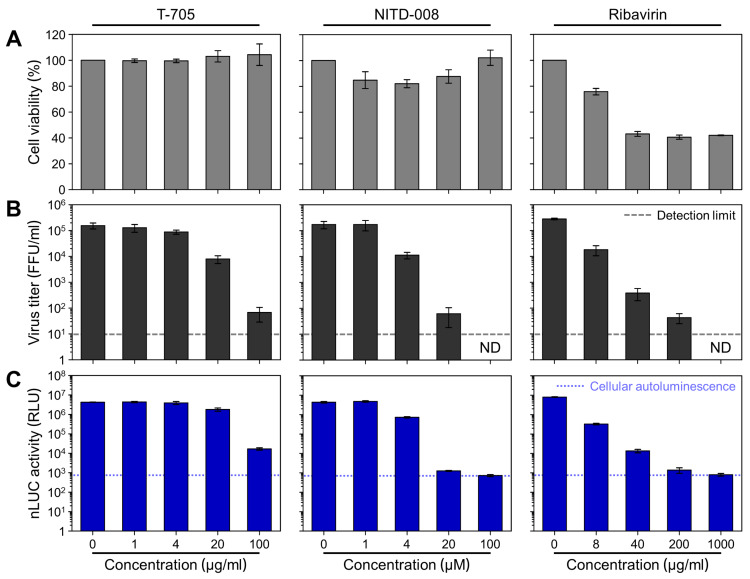
Application of rZIKV-nLUC for testing antiviral compounds. (**A**) Cell toxicity. BHK-21 cells were treated with T-705 (0, 1, 4, 20, and 100 μg/mL), NITD-008 (0, 1, 4, 20, and 100 μM), or ribavirin (0, 8, 40, 200, and 1000 μg/mL) for 48 h. At the end of the incubation, cell viability was measured using the CellTiter-Blue Cell Viability Assay. (**B**,**C**) Antiviral activity. BHK-21 cells were mock-infected or infected with rZIKV-nLUC at a MOI of 0.1 and treated with T-705, NITD-008, or ribavirin at one of the indicated concentrations for 48 h. Antiviral activity was analyzed with regard to both the level of extracellular virus production in culture supernatants using our α-NS3 antibody-based immunofocus assay (**B**) and the level of intracellular nLUC expression in cell lysates using the Nano-Glo Luciferase Assay (**C**). The data represent mean values, and the error bars show standard deviations. ND, Not detectable.

Our series of immunoblot analyses revealed both expected and unexpected results. As expected, in cells transfected with pZIKV-derived RNA and in those transfected with RNA derived from pZIKV-eGFP or pZIKV-nLUC, we observed a similar overall expression profile of the mature ZIKV proteins, together with related species that were presumed to represent cleavage intermediates or further cleavage/degradation products. Specifically, in all three cases, this viral protein expression profile was mainly defined by the following proteins, labeled on the basis of our previous report [12]: (1) the ~13-kDa C and at least one related smaller product of ~11 kDa (Figure 3A); (2) the ~9-kDa M, its ~24-kDa precursor prM, and notably three prM-related smaller products of 11–19 kDa (Figure 3B); (3) the ~56-kDa E and at least three related smaller products of 14, 26, and 45 kDa (Figure 3C); (4) the ~45-kDa NS1 (Figure 4A); (5) the ~14-kDa NS2B (Figure 4B); (6) the ~69-kDa NS3, its ~85-kDa cleavage intermediate NS2B-NS3 or NS3-NS4A/4A’, and multiple NS3-related smaller products of 33–60 kDa (Figure 4C); (7) the ~14-kDa NS4A’, a further-processed NS4A derivative lacking its C-terminal transmembrane domain, together with at least two minor proteins NS4A^p29^ and NS4AB^p35^ (Figure 4D); (8) the ~27-kDa NS4B and at least two related proteins NS4B^p11^ and NS4AB^p35^ (Figure 4E); and (9) the ~103-kDa NS5 (Figure 4F). To our surprise, however, we also noted two previously unrecognized larger forms of C (C^p14^, Figure 3A) and NS1 (NS1^p50^, Figure 4A), predominantly accumulated in cells transfected with RNA derived from pZIKV-eGFP or pZIKV-nLUC, but barely detectable in cells transfected with pZIKV-derived RNA; the nature of the increase in their molecular sizes is unknown. With respect to viral protein accumulation kinetics, as expected, there was a delay of ~24 h associated with the two reporter-encoding ZIKVs, which is in good agreement with the increase in their genome sizes.

### 3.4. Characterization of Reporter Gene Expression During ZIKV Genomic RNA Replication

We examined the expression of a fluorescent or luminescent reporter gene from both the RNAs derived from our reporter-encoding full-length ZIKV cDNA clones and the viruses recovered from them. First, we qualitatively determined the production of eGFP in BHK-21 cells at 40 h after RNA transfection (Figure 5A) and after virus infection (Figure 5B). In both experiments, confocal microscopy revealed strong green fluorescent signals in the nucleus and cytoplasm, with a notably brighter signal in the nucleus, not only in the cells transfected with pZIKV-eGFP-derived RNA but also in those infected with rZIKV-eGFP. The intracellular distribution of the fluorescent signals was consistent with the fact that the eGFP molecule has a calculated molecular size of ~29 kDa, small enough to be able to move between the nucleus and the cytoplasm by diffusion. As expected, such fluorescent signals were not observed in mock-transfected or mock-infected cells, nor were they detected in cells transfected with pZIKV-derived RNA or infected with rZIKV. Thus, our data show that eGFP is expressed from both the in vitro-synthesized RNA of pZIKV-eGFP and the viral genomic RNA of rZIKV-eGFP.

Next, we quantitatively evaluated the level of nLUC activity in BHK-21 cells over a 4-day period following RNA transfection (Figure 5C). To serve as a negative control for the replication-competent pZIKV-nLUC, we created its replication-incompetent counterpart, pZIKV-nLUC^POL−^, by substituting alanines for the two aspartic acid residues within the conserved GDD sequence motif located at the catalytic site of the NS5 polymerase domain [67]. In the lysate of cells transfected with pZIKV-nLUC^POL−^-derived RNA, the initial nLUC activity at 6 hpt was 3.0 × 10^4^ relative light units (RLU), representing the translation of the input RNA in the absence of viral RNA replication. This level then decreased slowly to 4.0 × 10^3^ RLU at 96 hpt. In the lysate of cells transfected with pZIKV-nLUC-derived RNA, on the other hand, the initial nLUC activity at 6 hpt was 3.6 × 10^4^ RLU, a level slightly higher than that obtained at the same time point from pZIKV-nLUC^POL−^-derived RNA. In this case, the level then increased rapidly to 7.3 × 10^6^ RLU by 48 hpt and peaked at 4.7 × 10^7^ RLU at 72 hpt. As expected, no nLUC activity above the background of cellular luminescence was detected in the lysate of mock-transfected cells or cells transfected with pZIKV-derived RNA. These results demonstrate that nLUC is expressed from the in vitro-synthesized RNA of pZIKV-nLUC in a manner dependent on viral RNA replication.

We also measured the activity of nLUC for 4 days after the infection of BHK-21 cells with rZIKV-nLUC to confirm the reporter expression from the incoming viral genomic RNA (Figure 5D). For this experiment, BHK-21 cells were mock-infected or infected with rZIKV-nLUC or rZIKV as a control at a MOI of 1. In the lysate of cells infected with rZIKV-nLUC, the initial nLUC activity at 6 h post-infection (hpi) was 7.3 × 10^3^ RLU, dropping temporarily to 3.8 × 10^3^ RLU at 12 hpi before substantially accumulating newly synthesized genomic RNAs, but then increasing sharply and peaking at 2.2 × 10^7^ RLU at 60 hpi. As expected, neither mock-infected nor rZIKV-infected cells exhibited any nLUC activity above the background level. Therefore, our results indicate that nLUC is expressed from the viral genomic RNA of rZIKV-nLUC and its expression level increases as the replication progresses.

### 3.5. Examination of the Viral Growth and Stability of Two Reporter-Expressing ZIKVs

We assessed the growth kinetics of our two reporter-expressing ZIKVs in BHK-21 cells following infection at a MOI of 1 with rZIKV-eGFP or rZIKV-nLUC, or with rZIKV for comparison; viral titers were determined by immunofocus assays on naïve BHK-21 cells by using a rabbit α-NS3 antiserum. During a 4-day incubation period, rZIKV initially displayed a short eclipse phase, with a titer of 3.5 × 10^3^ FFU/mL at 12 hpi; this phase was followed by a sharp burst phase, reaching a titer of 1.9 × 10^6^ FFU/mL by 24 hpi and peaking at 1.3 × 10^7^ FFU/mL at 48 hpi (Figure 6A). On the other hand, both rZIKV-eGFP and rZIKV-nLUC uniformly showed a delay of ~24 h in viral growth, with their eclipse phases lasting until 18 hpi and achieving their peak titers of 6.5–8.0 × 10^6^ FFU/mL at 72 hpi (Figure 6A). Thus, our data show that our two reporter-expressing ZIKVs grow to a high titer, but more slowly than their parent rZIKV.

During the analysis of viral growth kinetics, we also monitored the morphology of infectious foci developed by the progeny virions that were released over a period of 4 days from BHK-21 cells infected with rZIKV-eGFP, rZIKV-nLUC, or rZIKV. The homogenous, large foci generated by rZIKV remained unchanged for the entire 4 days (Figure 6B). However, the homogenous, tiny foci produced by rZIKV-eGFP and rZIKV-nLUC both exhibited marked visible changes, increasing in both size and heterogeneity over time, with the new foci becoming as large as those formed by rZIKV (Figure 6B). These phenotypic changes are indicative of genetic instability in the two reporter-expressing ZIKVs. Indeed, both rZIKV-eGFP and rZIKV-nLUC nearly lost their ability to express their respective reporter genes after five serial passages of each virus in BHK-21 cells at a MOI of 1 (data not shown). Therefore, our results indicate that our two reporter-expressing ZIKVs, directly recovered from their respective cDNA clones, contained their functional reporter expression cassettes; however, in both cases, the inserted reporter expression cassette became non-functional during subsequent propagation in cell culture.

### 3.6. Evaluation of the Antiviral Activity of Three Compounds Using rZIKV-nLUC

We utilized a stock of the unpassaged rZIKV-nLUC virus directly recovered from its functional cDNA clone to compare the in vitro anti-ZIKV activity of three different nucleoside analogs that can act as a substrate for the viral NS5 (Figure 7): (i) the purine analog T-705, also known as favipiravir (6-fluoro-3-hydroxy-2-pyrazinecarboxamide) [68,69,70,71,72]; (ii) the adenosine analog NITD-008 (7-deaza-2′-C-acetylene-adenosine) [73,74,75]; and (iii) the guanosine analog ribavirin (1-β-D-ribofuranosyl-1,2,4-triazole-3-carboxamide) [68,69,72,76]. To this end, BHK-21 cells were mock-infected or infected with rZIKV-nLUC at a MOI of 0.1 in a 96-well plate and incubated in the absence or presence of each nucleoside analog at four different concentrations that had little or only moderate cytotoxic effects on the cell line (Figure 7A). At 48 h after infection, culture supernatants were harvested to examine the production level of extracellular infectious virions and cell monolayers were lysed to analyze the expression level of intracellular nLUC as an indicator of viral RNA replication. We observed that all three nucleoside analogs exhibited a dose-dependent inhibition of the production of progeny virions in the presence of increasing amounts of each compound (Figure 7B). More importantly, this decrease in virus production was directly correlated with the reduction in nLUC expression (Figure 7C). It was also noted that treatment with NITD-008 at 100 μM or ribavirin at 1000 μg/mL equally produced a complete inhibition of viral RNA replication and consequently, virus production (Figure 7B,C). Taken together, these results demonstrate the feasibility of using reporter-expressing ZIKVs to facilitate the screening and testing of new antiviral drugs against this pathogen.

## 4. Discussion

In this report, we describe the development of two reporter-encoding full-length functional cDNA clones of ZIKV: pZIKV-eGFP, which encodes an enhanced green fluorescent protein, and pZIKV-nLUC, which encodes an improved luminescent NanoLuc luciferase. In both cases, transfection of ZIKV-susceptible BHK-21 cells with run-off RNA transcripts synthesized in vitro by SP6 RNA polymerase from the *Psr*I-linearized cDNA template resulted in the production of reporter-expressing ZIKVs, although the insertion of such a reporter expression cassette into the viral genome caused a delay in viral RNA replication, protein accumulation, and eventually, virus production when compared to parental ZIKV. Interestingly, a series of immunoblotting experiments with each of nine ZIKV region-specific polyclonal rabbit antisera identified two previously overlooked larger forms, each related to C and NS1, both of which accumulated to readily detectable levels in cells transfected with the RNA transcribed from pZIKV-eGFP or pZIKV-nLUC, but only to barely detectable levels in cells transfected with the parental pZIKV-derived RNA. We also employed our nLUC-expressing ZIKV as a platform to assess the antiviral activity of three nucleoside analogs, T-705, NITD-008, and ribavirin. Taken together, our two reporter-expressing ZIKVs and their functional cDNA clones provide useful molecular tools not only for the study of viral infection/replication, but also for the development of antiviral drugs [77,78].

We created two reporter-encoding full-length functional cDNA clones of ZIKV MR-766 based on a BAC plasmid [79], a single-copy vector that has enabled us to maintain a genetically unstable full-length ZIKV cDNA [12] and has been shown to minimize the destabilizing effects on other full-length flavivirus cDNAs [80] that occur during its amplification in *E. coli* with multiple-copy vectors [81,82,83,84,85]. Using our two reporter-encoding full-length ZIKV BAC clones, we produced reporter-expressing viruses by employing a single plasmid-based “RNA-launched” reverse genetic approach, which involved the transfection of infectious RNA transcripts synthesized from a full-length ZIKV cDNA that is flanked by a phage SP6 promoter at the 5′ end and a unique *Psr*I restriction site at the 3′ end for in vitro run-off transcription. Following RNA transfection, the infectious ZIKV RNAs, like the viral genomic RNA, directly underwent viral translation and RNA replication in the cytoplasm of the transfected cells. Several conceptually similar RNA-launched systems have previously been developed to construct a full-length functional ZIKV cDNA clone, based on a single- or low-copy vector. For these, a combination of a 5′ phage promoter (SP6 or T7) with either a 3′ unique recognition site for one of three restriction endonucleases (*Age*I, *Xho*I, and *Bss*HII) or a 3′ self-cleaving ribozyme sequence of hepatitis delta virus (HDVr) has been used [26,67,86,87,88,89]. These systems have been employed to generate recombinant ZIKVs expressing one of the following seven reporters: one of three fluorescent proteins (eGFP, mCherry, or turboFP635) or four luciferases (Photinus, Renilla, Luciola, or NanoLuc) [67,86,87], as well as replication-competent but propagation-deficient subgenomic replicons expressing one of two luciferases (Renilla or Gaussia) [67,87,89,90,91].

Although the system we describe in the present study is conceptually similar to all the other single plasmid-based RNA-launched systems reported previously, there are two key technical differences: (i) Our study produced reporter-expressing ZIKVs with an EMCV IRES-driven reporter gene expression cassette inserted downstream of the single ORF of the ZIKV genome. Therefore, the level of reporter gene expression depended on the actual number of viral genomic RNAs during the replication process, although its translation was controlled by the EMCV IRES element. In contrast, all the previous studies have introduced a particular reporter gene in-frame after a partial or complete sequence of the viral C protein that contains a *cis*-acting cyclization sequence required for viral RNA replication, followed in-frame by the foot-and-mouth disease virus (FMDV) 2A autoprotease sequence and then the entire ZIKV ORF that contains a functional or non-functional cyclization sequence within its C protein-coding region [67,86,87,89,90,91]. The resulting reporter-expressing ZIKVs therefore have a reporter-FMDV 2A gene segment placed in-frame upstream of the viral ORF under the control of its own 5′UTR. (ii) It is noteworthy that in our current study, for cDNA linearization, we applied the extremely rare-cutting restriction endonuclease *Psr*I (N_7_↓N_12_GAACN_6_TACN_12_↓N_7_), which cuts on both sides of its recognition sequence after any nucleotide. The application of *Psr*I is highly advantageous not only because it was far less likely to find a preexisting site(s) in the viral genome than were most site-specific restriction endonucleases, of which their recognition sequences are from four to eight bases long, but also because we could generate synthetic RNAs with the authentic 3′ end of the viral genome by run-off transcription of the *Psr*I-linearized full-length ZIKV cDNA. In the previously reported systems, however, a full-length functional ZIKV cDNA clone has been linearized either by using one of three six-base-recognizing classical type II endonucleases (*Age*I, *Xho*I, or *Bss*HII), all of which leave five non-viral extra nucleotides at the 3′ end of in vitro-transcribed full-length RNAs [26,67,87,88], or by using the ~85-nt self-cleaving ribozyme HDVr, which generates the authentic viral 3′ end [86,89], as was accomplished in our study by using the bipartite seven-base-recognizing unusual type IIB endonuclease *Psr*I. Creating the authentic 3′ end is of particular importance because this reaction has been shown to be critical for the production of infectious ZIKV RNAs [12].

As an alternative to the RNA-launched approach, a single plasmid-based “DNA-launched” approach has been developed to recover infectious ZIKVs from a cloned cDNA. This alternative process involves the transfection of a single- or multiple-copy vector carrying a full-length infectious ZIKV cDNA that is flanked at the 5′ end by an eukaryotic RNA polymerase II (Pol II)-dependent cytomegalovirus promoter and at the 3′ end by an HDVr sequence plus a simian virus 40-derived or bovine growth hormone gene-derived polyadenylation signal and transcription terminator [87,92,93,94]. In most cases, the full-length ZIKV cDNA has been engineered to include an artificial intron(s) to circumvent its genetic instability during propagation in bacteria [87,92,93]. Following DNA transfection, the infectious ZIKV cDNAs are initially transcribed in the nucleus of transfected cells by cellular RNA Pol II and the primary transcripts are processed and transported to the cytoplasm, where viral RNA replication takes place. This vector-oriented system has been modified to develop two non-vector-oriented systems: The first requires the transfection of a circular full-length ZIKV cDNA that is synthesized by a circular polymerase extension cloning method using a mixture of eight overlapping cDNA fragments [95]. The second requires the co-transfection of three or four overlapping cDNA fragments, which are eventually joined into a linear full-length ZIKV cDNA by spontaneous recombination in the transfected cell, although inefficiently [96,97]. Of these two new systems, the second one has been used to generate GFP-expressing ZIKVs by introducing the GFP-FMDV 2A gene segment in-frame, downstream of a partial sequence of the ZIKV C protein, followed in-frame by the entire ZIKV ORF [97].

Reporter-expressing viruses provide a powerful tool not only for the detection and analysis of viral replication, but also for the screening and testing of antiviral agents without specific labeling of viral components [77,78]. In this study, we utilized both the fluorescent eGFP and the luminescent nLUC as reporters for ZIKV because each reporter offers particular benefits that are useful for prospective applications. First, a fluorescent protein facilitates single-cell analysis by enabling the tracking of infected individual cells. One of the main applications of fluorescent protein-expressing ZIKVs is fluorescence-activated cell sorting (FACS) analysis, in which cells are separated using a flow cytometer based on the presence or absence of a fluorescent signal [98]. Recently, this method has been shown to be useful in testing potential antivirals against ZIKV, since the number of GFP-positive infected cells decreases when they are treated with a known flavivirus inhibitor such as ribavirin or interferon-β [97]. In the case of the luciferase reporter, the ability to quantify enzyme activity in a simple manner has led to its common use as a reporter in various flaviviruses to expedite the discovery process of novel antivirals. One of the primary means by which this discovery has been accomplished is through the optimization of a high-throughput assay with the use of luciferase-expressing ZIKV [86] and other flaviviruses (e.g., DENV and WNV) [99,100,101,102]. The present study took advantage of nLUC, an improved version of luciferase that can provide an advanced screening platform empowered by a higher reporter protein stability, a lower detection limit, and a broader dynamic range than traditional Photinus and Renilla luciferases [66]. In particular, the higher stability of nLUC was highlighted by our results described in this study, demonstrating that there were no significant decreases in its activity for the first 48 h after transfection of the replication-incompetent pZIKV-nLUC^POL−^-derived RNA into BHK-21 cells. By comparison, an analogous recombinant JEV expressing the Photinus luciferase developed by our laboratory exhibited a nearly 2-log decrease in luciferase activity over the same time span under identical experimental conditions [60]. This improved stability of nLUC offers a relatively long time window to more precisely quantify an inhibitory effect of potential antiviral compounds against ZIKV. In this study, we showed the usefulness of nLUC-expressing ZIKV in testing three nucleoside analogs in vitro as potential antivirals, which can act as a substrate for the ZIKV NS5. Given the fact that several recombinant viruses expressing a luciferase gene are often used for in vivo bioluminescence imaging to visualize the sites of viral infection and replication and monitor the dynamics of viral spread in live animals [77,103], this optical molecular imaging technique might also be a potential application for an improved version of our nLUC-expressing ZIKV with a higher genetic stability in future studies.

As is often the case with reporter-expressing flaviviruses [78,80], our results indicate that the insertion of a reporter gene into the genome of ZIKV led to an attenuation of viral replication and a decrease in genetic stability. The larger genomes resulted in slower replication which, in turn, created a selective pressure for the reporter-expressing ZIKVs to delete all or part of the inserted reporter gene that is unnecessary for viral replication. As noted previously [87,97], we observed signs of this deletion occurring after serial passage of rZIKV-eGFP and rZIKV-nLUC, with their infectious foci formed on monolayers of BHK-21 cells growing in size and becoming increasingly heterogeneous over time. Despite the long-term genetic instability of their viral genomes, we were able to recover reporter-expressing ZIKVs for the first 4 days after transfection of infectious RNAs transcribed in vitro from the respective cDNAs. Our choice to insert a reporter gene after the stop codon of the ZIKV ORF was based on our previous work with JEV showing that such placement had less of a negative impact on viral replication than did the placement of a reporter gene before the start codon of the viral ORF [60,104]; a similar strategy has also been shown to be effective in generating reporter-expressing WNV [105,106] and DENV [107,108]. Interestingly, however, it appears that our eGFP- and nLUC-expressing ZIKVs described in the present study showed greater levels of genetic instability than have the similar GFP- and luciferase-expressing JEVs previously developed by our laboratory [60,104]. An alternative approach commonly employed in the development of reporter-expressing flaviviruses is to place a reporter gene upstream of the viral full-length ORF, such as with WNV [109], DENV [99,110,111,112], and ZIKV [67,86,87,97]. In all these cases, reporter-expressing flaviviruses are genetically unstable, eventually losing the ability to express the reporter gene. Further work, including new approaches, is necessary to produce genetically stable reporter-expressing ZIKVs and other flaviviruses [113,114,115,116].

For both the eGFP- and nLUC-expressing ZIKVs we generated in this study, the translation of each reporter gene was driven by an IRES element. Of several known viral IRESs, we chose to use an EMCV IRES, not only because it has a stronger activity in terms of internal ribosomal entry than do other IRESs, including those from poliovirus, rhinovirus, foot-and-mouth disease virus, and hepatitis A and C viruses, but also because it is biologically functional in a variety of cell types, unlike those from poliovirus and rhinovirus [117,118]. Since the 619-nt version of the EMCV IRES that we used in the present study is quite long, it significantly increased the size of the viral genome and decreased the efficiency of viral replication. In the future, as a way of improving the genetic instability of our reporter-expressing ZIKVs, we can consider utilizing a shorter version of the EMCV IRES that contains only an ~450-nt core sequence for IRES function or employing one of the shorter IRESs, such as those from picornaviruses (~450 nt), hepatitis C virus (~350 nt), and cricket paralysis virus (~190 nt) [119,120]. Interestingly, a recent study has shown that the relatively short 5′UTR of the genomic RNA of two flaviviruses, i.e., DENV (~100 nt) and ZIKV (~110 nt), has IRES activity capable of directing the cap-independent internal initiation of translation [121]. Because of its short length and IRES activity, this 5′UTR would be a good candidate for the construction of IRES-driven reporter-expressing recombinant ZIKVs to improve our system.

Our reporter-expressing ZIKVs were developed as a bicistronic expression vector by keeping the single viral ORF under the cap-dependent translational control of its own 5′UTR as the first cistron and inserting the eGFP or nLUC gene under the cap-independent translational control of EMCV IRES as the second cistron at the end of the viral ORF. The insertion of such a reporter gene expression cassette into the viral genome increased the genome length, which led to a decrease in viral RNA replication. In a study using a similar IRES-mediated bicistronic expression vector designed for co-expression of two reporter genes, an inverse correlation has been demonstrated between the expression of one reporter and the other, suggesting a competition between the two coding sequences during translation [122]. Based on this previous study, we can expect that the high expression of the viral ORF will lower the expression of a reporter gene and vice versa. In addition, this earlier study also found that the translational competition is dependent on the size of the two genes [122]. In the present study, therefore, the viral ORF has a competitive disadvantage relative to the reporter gene, which inevitably leads to a decreased expression of viral proteins when compared to the same virus without a reporter gene. As a consequence, the lower replicative ability of our reporter-expressing ZIKVs is likely not only a result of the longer genome length, but may also be linked to the lower expression of viral proteins resulting from the presence of a reporter gene.

Immunoblot analysis revealed that the viral protein accumulation profiles generated from each of the eGFP- and nLUC-expressing ZIKV RNAs were similar to those produced from the parental ZIKV RNA with no reporter gene expression cassette, which is in agreement with our previous work [12]. The panel of nine ZIKV antigen-specific polyclonal rabbit antisera that we employed in this study detected all of the expected viral proteins for the parental and reporter-expressing ZIKV RNAs, but the kinetics of protein accumulation was delayed by ~24 h in the latter case. This delay was in line with the reduced viral growth rates and focus sizes. Beyond these similarities, we also found that immunoblotting with the α-C and α-NS1 antisera revealed a significant accumulation of two previously undescribed protein species (C^p14^ and NS1^p50^) expressed from each of the two reporter-expressing ZIKV RNAs. Although the nature of these two proteins is unknown, our data enable us to make several inferences about their identity: (i) The fact that C^p14^ and NS1^p50^ were strongly stained in cells harboring eGFP- or nLUC-expressing ZIKV RNA suggests that their accumulation is a result of changes caused by the insertion of a reporter gene expression cassette into the viral genome, not by the presence of either eGFP or nLUC specifically. (ii) It is unlikely that C^p14^ and NS1^p50^ correspond to any unknown degradation products of C and NS1, respectively, because C^p14^ (~14 kDa) is larger than C (~13 kDa) and NS1^p50^ (~50 kDa) is larger than NS1 (~45 kDa). (iii) C^p14^ cannot be the result of a non-canonical cleavage at the C-prM junction because there were no unexpected or altered protein bands present on the immunoblot stained with the α-M antiserum, which would be detectable in the event of alternate processing at the C-prM junction of the viral polyprotein. The same possibility of any non-canonical cleavage at the NS1-NS2A junction cannot be ruled out in the case of NS1^p50^ because there is no antibody currently available to detect ZIKV NS2A [12]. (iv) It is less likely that NS1^p50^ corresponds to NS1′, a C-terminally extended variant of NS1 that results from a -1 ribosomal frameshift occurring at codons 8–9 of NS2A in JEV and WNV infection [63,123,124,125], because ZIKV lacks two genetic elements, a slippery sequence and a predicted pseudoknot, required for the frameshifting (see Supplementary Figure S1). We therefore speculate that C^p14^ and NS1^p50^ are the result of unknown post-translational modifications of C and NS1, respectively. Given that both C^p14^ and NS1^p50^ were faintly detectable in cells harboring the parental ZIKV RNA, it is possible that the slow replication of eGFP- and nLUC-expressing ZIKV RNAs and the resulting delay in virus-induced cell death may allow for the accumulation of these two previously unrecognized viral proteins.

In sum, the present study presents the development, characterization, and application of two reporter-expressing ZIKVs. These recombinant viruses are replication-competent, expressing the fluorescent eGFP or luminescent nLUC protein in a manner dependent on viral RNA replication. Therefore, these recombinant viruses represent useful additions to the molecular tools currently available to the ZIKV research community, facilitating the advancement of our understanding of ZIKV infection/replication. Also, our two reporter-expressing ZIKVs can be instrumental in the development of novel antiviral agents.

## Figures and Tables

**Table 1:** Oligonucleotide primers used for the construction of reporter-encoding full-length ZIKV cDNA clones.

Oligonucleotide	Sequence (5′→3′)	Polarity
ORF3f	AAACGGACCGGGATCCCTTATAGGGCACAGAC	Sense
ORF3r	TTTGAATTCACACTAAAATTGGTGCTTACAA	Antisense
3UTRf	AAAATGCATGCACCAATTTTAGTGTTGTCAGG	Sense
3UTRr	TTTCCGCGGAGGGCGGCCGCGTATGTCGCGTT	Antisense
FORf	GTGGAAGGAGCTGGGGAAACGCAAGCGGCC	Sense
POLr	TGGCTTCACAACGCAGGCAGCTCCACTGACCGCCA	Antisense
POLf	TGGCGGTCAGTGGAGCTGCCTGCGTTGTGAAGCCA	Sense
REVr	CCAAGTGGTGCGGGGTCTGTGCCCTATAAG	Antisense
EGFPf	ACAACCATGGTGAGCAAGGGCGAGGAGCTGTT	Sense
EGFPr	TGCATGCATTCATTAACCGTCGACTGCAGAATT	Antisense

## Supplementary Materials

The following is available online at https://www.mdpi.com/1999-4915/12/5/572/s1, Figure S1: ZIKV lacks the ribosomal frameshift signal directing the expression of NS1′.
