# Supplementary material for: Development, Characterization, and Application of Two Reporter-Expressing Recombinant Zika Viruses
Source: Viruses. 2020 May 22;12(5):572. doi: 10.3390/v12050572 (PMC7290298; doi:10.3390/v12050572)
Supplement: Supplementary file 1 [file viruses-12-00572-s001.pdf]

*Supplementary Materials*

# Development, Characterization, and Application of Two Reporter-Expressing Recombinant Zika Viruses


**Sang-Im Yun[1], Byung-Hak Song[1], Michael E. Woolley[1], Jordan C. Frank[1], Justin G. Julander[1,2] and Young-Min Lee[1,3,*]**

[1] Department of Animal, Dairy, and Veterinary Sciences, College of Agriculture and Applied Sciences, Utah State University, Logan, UT 84322, USA; sangim.yun@usu.edu (S.-I.Y.); byunghak.song@aggiemail.usu.edu (B.-H.S.); michaeleverettwoolley@gmail.com (M.E.W.); jc.frank@aggiemail.usu.edu (J.C.F.); justin.julander@usu.edu (J.G.J.)

[2] Institute for Antiviral Research, Utah State University, Logan, UT 84322, USA

[3] Veterinary Diagnostics and Infectious Diseases, Utah Science Technology and Research, Utah State University, Logan, UT 84341, USA

* Correspondence: youngmin.lee@usu.edu; Tel.: +1-435-797-9667


## Supplemental Figure S1

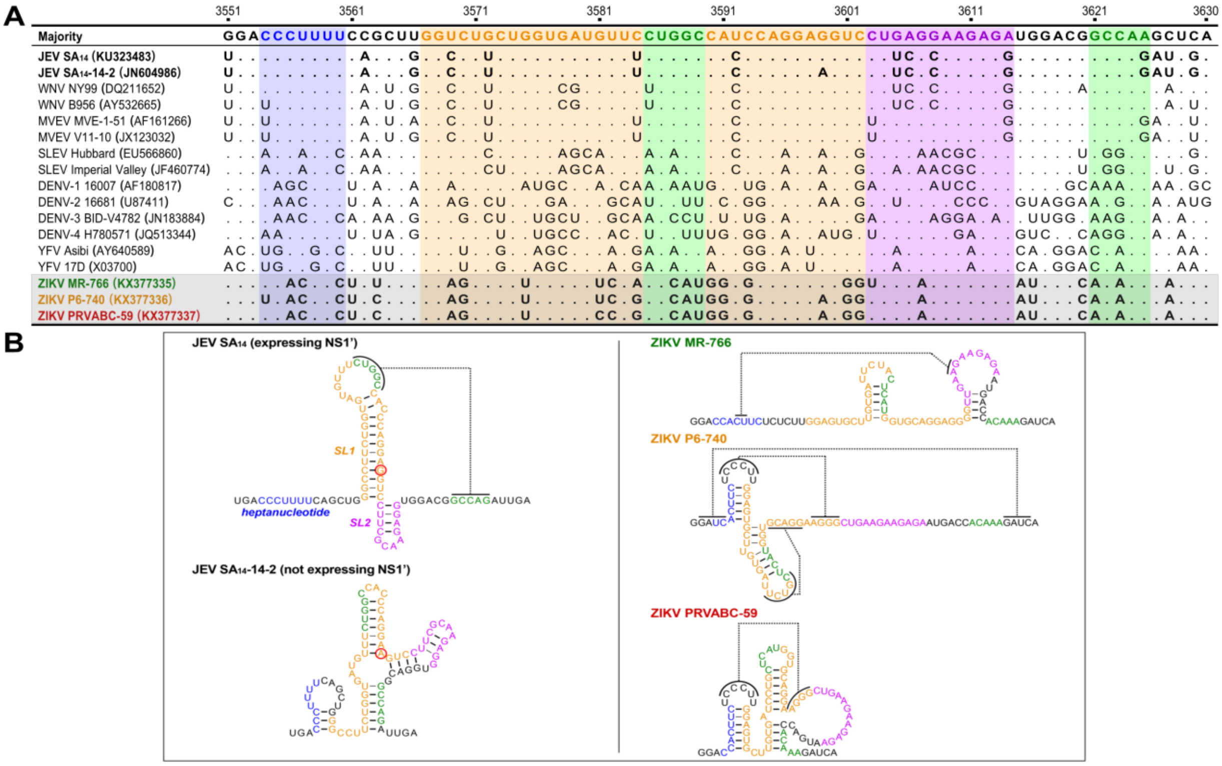


**Figure S1.** ZIKV lacks the ribosomal frameshift signal directing the expression of NS1′. (**A**) Nucleotide sequence alignment for seven major mosquito-borne flaviviruses (17 strains total). The consensus sequence is shown on top, and residues that match the consensus are hidden as dots to emphasize residues that differ from the consensus. (**B**) Predicted RNA folding involved in JEV NS1′ frameshifting and its ZIKV counterpart. RNA secondary structures with pseudoknots were predicted using the IPknot program. Highlighted are the primary sequences and secondary structures important for the expression of JEV NS1′: the heptanucleotide slippery sequence (blue), stem-loop 1 (SL1, orange), stem-loop 2 (SL2, magenta) and pseudoknot base-pairing (green). Also indicated is the silent point mutation $G^{3599}A$ (red circle) that is sufficient to abolish the synthesis of JEV $SA_{14}$ NS1′.
